# Retraction: Relative age effect: beyond the youth phenomenon

**DOI:** 10.1136/bmjsem-2020-000857ret

**Published:** 2024-03-26

Joyner PW, Lewis J, Mallon WJ, *et al.* Relative age effect: beyond the youth phenomenon. *BMJ Open Sport & Exercise Medicine* 2020;6:e000857. doi: 10.1136/bmjsem-2020-000857

This article has been retracted by the journal due to redundant publication.

The journal was made aware of an article^1^ first published in the *American Journal of Lifestyle Medicine* in 2017 with a similar author list to this article. On review, it was found that both articles present the same study and the same data is used. The authors were contacted for comment. They confirmed that the study is the same and apologised.

As the article in *BMJ Open Sports & Exercise Medicine* was published second, the journal is retracting the article in line with the Committee on Publication Ethics’ (COPE) guidelines regarding redundant publication.

The *American Journal of Lifestyle Medicine* has been notified of this retraction.
